# Association of polymorphisms in the MAFB gene and the risk of coronary artery disease and ischemic stroke: a case–control study

**DOI:** 10.1186/s12944-015-0078-2

**Published:** 2015-07-25

**Authors:** Qian Yang, Rui-Xing Yin, Yi-Jiang Zhou, Xiao-Li Cao, Tao Guo, Wu-Xian Chen

**Affiliations:** Department of Cardiology, Institute of Cardiovascular Diseases, the First Affiliated Hospital, Guangxi Medical University, 22 Shuangyong Road, Nanning, 530021 Guangxi People’s Republic of China

**Keywords:** V-maf avian musculoaponeurotic fibrosarcoma oncogene homolog B (MAFB) gene, Single nucleotide polymorphism, Coronary artery disease, Ischemic stroke, Lipids

## Abstract

**Background:**

The v-maf avian musculoaponeurotic fibrosarcoma oncogene homolog B gene (*MAFB*) has been associated with serum lipid levels in the Eurpean population, but little is known about such association in the Chinese population or in atherosclerosis-related patients. Therefore, the purpose of the present study was to assess the association of the single nucleotide polymorphisms (SNPs) in the *MAFB* and serum lipid levels and the risk of coronary artery disease (CAD) and ischemic stroke (IS) in the Chinese population.

**Methods:**

A total of 1,065 unrelated patients (CAD, 525 and IS, 540) and 539 healthy controls were recruited in this study. Genotypes of the *MAFB* rs2902940 and rs6102059 SNPs were determined by the Snapshot technology platform.

**Results:**

The rs2902940AA genotype was associated with an increased risk of CAD (adjusted OR = 1.63, 95 % CI = 1.07–2.48, *P* = 0.023) and IS (adjusted OR = 1.69, 95 % CI = 1.09–2.61, *P* = 0.017). The rs2902940GA/AA genotypes were also associated with an increased risk of CAD (adjusted OR = 1.56, 95 % CI = 1.04–2.32, *P* = 0.030 for GA/AA *vs*. GG) and IS (adjusted OR = 1.72, 95 % CI = 1.14–2.60, *P* = 0.010 for GA/AA *vs*. GG). Significant interactions were observed only in those with higher body mass index (BMI), hypertension and diabetes (*P* < 0.05). The subjects with rs2902940GA/AA genotypes in controls had lower serum ApoAI levels than the subjects with GG genotype (*P* = 0.024).

**Conclusions:**

The rs2902940A allele carriers in the *MAFB* conferred a decreased serum ApoAI level in controls and an increased risk of CAD and IS. The rs2902940GA/AA genotypes interacted with higher BMI, hypertension and diabetes to contribute the risk of CAD and IS.

## Background

Both coronary artery disease (CAD) and ischemic stroke (IS) have been recognized as widespread causes of death and disability for more than a century [1–3]. According to the Global Burden of Disease Study, cardiovascular and circulatory diseases accounted for 11.8 % of global DALYs (disability-adjusted life years), mainly comprising ischemic heart disease (5.2 %), haemorrhagic stroke (2.5 %), IS (1.6 %), and hypertensive heart disease (0.6 %) [2]. One of the leading pathophysiology mechanisms is atherosclerosis [4], which is a chronic process characterized by the deposition of excessive cholesterol in the arterial intima [5]. Elevated serum levels of total cholesterol (TC), triglyceride (TG), low-density lipoprotein cholesterol (LDL-C), and apolipoprotein (Apo) B, or low levels of high-density lipoprotein cholesterol (HDL-C) and ApoAI are among the most important risk factors for atherosclerotic vascular diseases [6–11]. Genetic and environmental factors interact to determine the lipid metabolism. Recently genome-wide association studies (GWAS), which could display genetic contribution to atherosclerosis, have identified multiple lipid-related loci and provided valuable information to develop novel therapeutic interventions for atherosclerosis [12–14].

The v-maf avian musculoaponeurotic fibrosarcoma oncogene homolog B gene (*MAFB*) is one of the novel genes reportedly to be associated with serum lipids in the Eurpean population [13, 14]. *MAFB* is localized on chromosome 20q11.2-q13.1 and belongs to the large Maf transcription factors, which contain N-terminal transactivation and C-terminal basic leucine-zipper (bZIP) deoxyribonucleic acid (DNA) binding domains [15]. *MAFB* is a transcription factor that induces myelomonocytic differentiation. It is expressed specifically in the myeloid lineage of the hematopoietic system and is up-regulated successively during myeloid differentiation from multipotent progenitors to macrophages [15, 16]. MAFB plays an important role in the terminal differentiation of macrophages. Macrophages are essential in the occurrence and development of atherosclerosis [17–20]. For the formation of atherosclerotic plaques, macrophages are involved in the removal of lipids and tissue debris and make a critical contribution to tissue damage and wall remodeling [21]. When inflow and esterification of cholesterol increased and/or its outflow decreased, the macrophages are ultimately transformed into lipid-laden foam cells, the prototypical cells in the atherosclerotic plaque [17, 22]. In an earlier study, *MAFB* was found to promote hyperlipidemic atherosclerosis by suppressing foam-cell apoptosis [19]. Moreover, in recent meta-analyses, the two loci in the *MAFB* were reported to associate with serum lipid metabolism in the Eurpean population [13, 14]. One was the rs2902940 single nucleotide polymorphism (SNP), which an A/G variation on human chromosome 20 [13]. Another was the rs6102059 SNP, which a C/T variation also on human chromosome 20 [14]. The minor alleles of the two SNPs had been associated with the levels of serum LDL-C. However, little is known about such association in the Chinese population. In addition, the association of the *MAFB* SNPs with atherosclerosis-related diseases has never been detected before. Therefore, the purpose of the present study was to assess the association of the rs2902940 and rs6102059 SNPs in the *MAFB* and serum lipid levels and the risk of CAD and IS in the Chinese population.

## Results

### Characteristics of the subjects

The clinical characteristics of the patients and healthy controls are shown in Table 1. The differences in age, gender, serum LDL-C and ApoB levels, and the percentages of subjects who had hyperlipidemia and smoked cigarettes were not significant between the control and patient groups (*P* > 0.05). As compared with the control group, more patients in CAD and IS groups had type 2 diabetes mellitus (T2DM), hypertension and also had higher body mass index (BMI), systolic blood pressure, diastolic blood pressure, serum TG levels (*P* < 0.05). In addition, compared with the control group, the IS patients had higher levels of pulse pressure and lower levels of serum TC, HDL-C, ApoAI, the ApoAI/ApoB ratio and the percentages of subjects who consumed alcohol (*P* < 0.05). The CAD patients, in contrast, there was no difference in the levels of pulse pressure, serum TC, HDL-C, ApoAI, the ApoAI/ApoB ratio and the percentages of subjects who consumed alcohol (*P* > 0.05). In comparison with CAD patients, the IS patients had higher blood pressure and serum TG levels, and lower the ApoAI/ApoB ratio and serum HDL-C levels (*P* < 0.05). The frequency of using lipid-lowing drugs was 0 % in the controls, 32.8 % in the CAD patients, and 27.8 % in the IS patients, respectively.

**Table 1:** General characteristics and serum lipid levels between the controls and patients

Characteristic	Controls (*n* = 539)	Patients		*P*		
		CAD (*n* = 525)	IS (*n* = 540)	*P*_1_	*P*_*2*_	*P*_*3*_
Male/female	374/165	383/142	390/150	0.223	0.316	0.837
Age, years	63.08 ± 10.76	62.27 ± 10.93	62.81 ± 12.36	0.222	0.702	0.448
Body mass index, kg/m^2^	22.84 ± 3.51	23.29 ± 3.33	24.75 ± 5.18	0.033	0.048	0.135
Systolic blood pressure, mmHg	133.18 ± 22.95	137.00 ± 23.28	147.54 ± 21.98	0.007	<0.001	<0.001
Diastolic blood pressure, mmHg	79.78 ± 13.03	81.82 ± 14.13	83.71 ± 12.92	0.014	<0.001	0.023
Pulse pressure, mmHg	54.38 ± 17.94	55.86 ± 18.19	63.80 ± 17.98	0.185	<0.001	<0.001
Cigarette smoking, n (%)	220(40.8)	233(44.4)	226(41.9)	0.264	0.757	0.421
Alcohol consumption, n (%)	232(43.0)	220(41.9)	198(36.7)	0.710	0.035	0.090
Total cholesterol, mmol/L	4.70 ± 1.18	4.61 ± 1.18	4.52 ± 1.15	0.217	0.014	0.232
Triglyceride, mmol/L	1.08(0.81)	1.21(0.80)	1.36(0.93)	0.026	<0.001	<0.001
HDL-C, mmol/L	1.48 ± 0.49	1.45 ± 0.51	1.23 ± 0.40	0.267	<0.001	<0.001
LDL-C, mmol/L	2.78 ± 0.93	2.71 ± 0.90	2.68 ± 0.90	0.255	0.083	0.555
Apolipoprotein (Apo) AI, g/L	1.17 ± 0.32	1.15 ± 0.29	1.02 ± 0.22	0.200	<0.001	0.288
ApoB, g/L	0.89 ± 0.23	0.90 ± 0.25	0.89 ± 0.25	0.574	0.882	0.692
ApoAI/ApoB	1.45 ± 1.76	1.37 ± 0.66	1.17 ± 0.61	0.366	0.001	<0.001
Type 2 diabetes mellitus, n (%)	75(13.9)	117(22.3)	116(21.5)	<0.001	0.001	0.751
Hypertension, n (%)	174(32.3)	266(50.7)	289(53.5)	<0.001	<0.001	0.107
Hyperlipidemia, n (%)	198(36.7)	210(40.0)	212(39.3)	0.284	0.415	0.851
Using lipid-lowing drugs, n (%)	0(0)	172(32.8)	150(27.8)	<0.001	<0.001	0.077

*CAD* coronary artery disease, *IS* ischemic stroke, *HDL*-*C* high-density lipoprotein cholesterol, *LDL*-*C* low-density lipoprotein cholesterol

*P*_1_: comparison of CAD and controls; *P*_2_: comparison of IS and controls; *P*_3_: comparison of CAD and IS

### Genotypic and allelic frequencies

The genotypic and allelic frequencies of *MAFB* rs2902940 and rs6102059 SNPs are presented in Table 2. The genotype distribution of the two SNPs was concordant with the Hardy-Weinberg proportions in both cases and controls. For the rs2902940 SNP, the frequency of the G and A alleles was 34.6 and 65.4 % in the controls, 29.9 and 70.1 % in the CAD patients, and 31.1 and 68.9 % in the IS patients; respectively. The frequency of the GG, GA and AA genotypes was 13.7, 41.8 and 44.5 % in the controls, 9.0, 41.9 and 49.1 % in the CAD patients, and 8.7, 44.8 and 46.5 % in the IS patients; respectively. The genotype frequencies were different between the control and patient groups (*P* < 0.05), and the allele frequency was also different between the controls and CAD patients (*P* < 0.05).

**Table 2:** Genotypic and allelic frequencies and the risk of CAD and IS

Genotype or allele	Control (%)	CAD (%)	IS (%)	CAD		IS	
	*n* = 539	*n* = 525	*n* = 540	OR (95 % CI)	*P*	OR (95 % CI)	*P*
rs2902940							
GG	74(13.7)	47(9.0)	47(8.7)	1		1	
GA	225(41.8)	220(41.9)	242(44.8)	1.48(0.97–2.26)	0.071	1.76(1.14–2.71)	0.011
AA	240(44.5)	258(49.1)	251(46.5)	1.63(1.07–2.48)	0.023	1.69(1.09–2.61)	0.017
*P*		0.038	0.032				
G	373(34.6)	314(29.9)	336(31.1)	1			
A	705(65.4)	736(70.1)	744(68.9)	1.22(1.01–1.48)	0.036	1.18(0.98–1.41)	0.078
*P*		0.021	0.084				
HWE(*P*)	0.071	0.992	0.290				
GG	74(13.7)	47(9.0)	47(8.7)	1		1	
GA + AA	465(86.3)	478(91.0)	493(91.3)	1.56(1.04–2.32)	0.030	1.72(1.14–2.60)	0.010
*P*		0.014	0.009				
AA	243(45.1)	258(49.1)	251(46.5)	1		1	
GA + GG	296(54.9)	267(50.9)	289(53.5)	0.85(0.66–1.09)	0.208	0.93(0.72–1.20)	0.559
*P*		0.185	0.645				
rs6102059							
CC	121(22.4)	123(23.4)	108(20.0)	1		1	
CT	249(46.2)	261(49.7)	280(51.9)	1.01(0.73–1.39)	0.958	1.19(0.85–1.66)	0.303
TT	169(31.4)	141(26.9)	152(28.1)	0.78(0.55–1.11)	0.170	0.93(0.64–1.33)	0.676
*P*		0.267	0.178				
C	491(44.5)	507(48.3)	496(45.9)				
T	587(54.5)	543(51.7)	584(54.1)	0.90(0.76–1.07)	0.233	0.99(0.83–1.17)	0.891
*P*		0.206	0.860				
HWE(*P*)	0.111	0.917	0.307				
CC	129(23.9)	123(23.4)	108(20.0)	1		1	
CT + TT	410(76.1)	402(76.6)	432(80.0)	0.96(0.71–1.28)	0.764	1.13(0.83–1.54)	0.443
*P*		0.847	0.119				
TT	169(31.4)	141(26.9)	152(28.1)	1		1	
CT + CC	370(68.6)	384(73.1)	388(71.9)	1.29(0.98–1.69)	0.074	1.22(0.93–1.61)	0.155
*P*		0.107	0.249				

*HWE* Hardy-Weinberg equilibrium, *CAD* coronary artery disease, *IS* ischemic stroke

For the rs6102059 SNP, the frequency of the C and T alleles was 44.5 and 54.5 % in the controls, 48.3 and 51.7 % in the CAD patients, and 45.9 and 54.1 % in the IS patients; respectively. The frequency of the CC, CT and TT genotypes was 22.4, 46.2 and 31.4 % in the controls, 23.4, 49.7 and 26.9 % in the CAD patients, and 20.0, 51.9 and 28.1 % in the IS patients; respectively. The genotypic and allelic frequencies were not significantly different between the controls and patients. No obvious linkage disequilibrium (LD) was noted between the two SNPs (*D*’ = 0.282; *r*^2^ = 0.041). Hence, we did not perform haplotype analyses.

### *MAFB* SNPs and the risk of CAD and IS

For the rs2902940 SNP, the A allele and the AA genotype were associated with an increased risk of CAD (adjusted Odds ratio (OR) = 1.22, 95 % confidence interval (CI) = 1.01–1.48, *P* = 0.036 and adjusted OR = 1.63, 95 % CI = 1.07–2.48, *P* = 0.023; respectively). The GA and AA genotypes were associated with an increased risk of IS (adjusted OR = 1.76, 95 % CI = 1.14–2.71, *P* = 0.011 and adjusted OR = 1.69, 95 % CI = 1.09–2.61, *P* = 0.017; respectively). The GA/AA genotype was also associated with an increased risk of CAD (adjusted OR = 1.56, 95 % CI = 1.04–2.32, *P* = 0.030 for GA/AA *vs*. GG) and IS (adjusted OR = 1.72, 95 % CI = 1.14–2.60, *P* = 0.010 for GA/AA *vs*. GG). Stratified analysis showed an increased risk of CAD and IS in subjects with the GA/AA genotype, mainly in those who belonged to one of the following subgroups: males (adjusted OR = 1.76, 95 % CI = 1.11–2.80, *P* = 0.016 for CAD and adjusted OR = 1.83, 95 % CI = 1.14–2.93, *P* = 0.012 for IS); aged ≤ 60 (adjusted OR = 2.28, 95 % CI = 1.16–4.49, *P* = 0.017 for CAD and adjusted OR = 2.76, 95 % CI = 1.39–5.41, *P* = 0.004 for IS); drinkers (adjusted OR = 1.96, 95 % CI = 1.02–3.78, *P* = 0.044 for CAD and adjusted OR = 2.53, 95 % CI = 1.26–5.07, *P* = 0.009 for IS); high BMI (adjusted OR = 2.77, 95 % CI = 1.38–5.57, *P* = 0.004 for CAD and adjusted OR = 2.73, 95 % CI = 1.14–5.33, *P* = 0.003 for IS); hypertension (adjusted OR = 2.11, 95 % CI = 1.09–4.08, *P* = 0.027 for CAD and adjusted OR = 2.10, 95 % CI = 1.12–3.92, *P* = 0.020 for IS); and diabetes (adjusted OR = 3.67, 95 % CI = 1.76–7.67, *P* = 0.001 for CAD and adjusted OR = 3.22, 95 % CI = 1.47–7.05, *P* = 0.003 for IS). In addition, there was an increased risk of IS in subjects with the GA/AA genotype, also in smokers (adjusted OR = 2.72, 95 % CI = 1.40–5.31, *P* = 0.003); and those with normal blood lipids (adjusted OR = 2.39, 95 % CI = 1.33–4.29, *P* = 0.004). Significant interactions were observed only in those with BMI > 24 kg/m^2^, hypertension and diabetes. No significant association was found between the rs6102059 SNP and the risk of CAD and IS (Tables 2 and 3).

**Table 3:** Stratified analysis between rs2902940 genotype and the risk of CAD and IS

Factors	CAD				IS			
	OR (95 % CI)			*P*_interaction_	OR (95 % CI)			*P*_interaction_
	GG	GA + AA	*P*		GG	GA + AA	*P*	
Gender								
Male	1	1.76(1.11–2.80)	0.016		1	1.83(1.14–2.93)	0.012	
Female	1	0.82(0.33–2.01)	0.658	0.366	1	1.39(0.50–3.89)	0.531	0.229
Age, year								
≤60	1	2.28(1.16–4.49)	0.017		1	2.76(1.39–5.41)	0.004	
>60	1	1.21(0.73–2.01)	0.460	0.326	1	1.39(0.82–2.36)	0.225	0.272
BMI, kg/m^2^								
≤24	1	1.17(0.70–1.95)	0.549		1	1.46(0.85–2.51)	0.171	
>24	1	2.77(1.38–5.57)	0.004	0.002	1	2.73(1.40–5.33)	0.003	0.001
Smoking								
No	1	1.35(0.75–2.43)	0.313		1	1.17(0.67–2.03)	0.578	
Yes	1	1.81(0.97–3.37)	0.061	0.073	1	2.72(1.40–5.31)	0.003	0.064
Alcohol								
No		1.18(0.68–2.05)	0.552		1	1.41(0.83–2.42)	0.207	
Yes		1.96(1.02–3.78)	0.044	0.904	1	2.53(1.26–5.07)	0.009	0.113
Hypertension								
No	1	1.29(0.75–2.23)	0.360		1	1.57(0.88–2.78)	0.126	
Yes	1	2.11(1.09–4.08)	0.027	<0.001	1	2.10(1.12–3.92)	0.020	<0.001
Hyperlipidemia								
No	1	1.50(0.91–2.49)	0.112		1	2.39(1.33–4.29)	0.004	
Yes	1	1.73(0.87–3.43)	0.118	0.082	1	1.33(0.70–2.51)	0.388	0.321
T2DM								
No	1	1.57(0.90–2.73)	0.110		1	1.62(0.93–2.82)	0.090	
Yes	1	3.67(1.76–7.67)	0.001	<0.001	1	3.22(1.47–7.05)	0.003	0.002

*CAD* coronary artery disease, *IS* ischemic stroke, *T2DM* type 2 diabetes mellitus

### *MAFB* SNPs and the angiographic severity of CAD

As shown in Table 4, there were no significant effects of the rs2902940 and rs6102059 SNPs on the angiographic severity of CAD in different genetic models (*P* > 0.05).

**Table 4:** Effect of the *MAFB* SNPs on angiographic severity of CAD

Locus/Genotype	Angiographic severity of CAD	
	OR (95 % CI)	*P*
rs2902940		
GG	1	
GA	0.93(0.46–1.89)	0.833
AA	1.09(0.54–2.20)	0.811
GG	1	
GA + AA	1.01(0.51–1.99)	0.975
rs6102059		
CC	1	
CT	1.08(0.67–1.76)	0.750
TT	0.89(0.52–1.53)	0.675
CC	1	
CT + TT	1.01(0.64–1.59)	0.964

*CAD* coronary artery disease

### Related risk factors for CAD and IS

Multivariate logistic analysis showed that the incidence of CAD positively correlated with hypertension and diabetes, and the incidence of IS positively correlated with hypertension, hyperlipidemia and negatively correlated with alcohol consumption (*P* < 0.05, Table 5).

**Table 5:** The relative risk factors for CAD and IS

Relative factors	CAD		IS	
	OR (95 % CI)	*P*	OR (95 % CI)	*P*
Male	1		1	
Female	0.80(0.60–1.06)	0.118	0.84(0.63–1.31)	0.254
Aged ≤ 60 year	1		1	
Aged > 60 year	0.78(0.61–1.02)	0.073	0.78(0.59–1.01)	0.062
BMI ≤ 24 kg/m^2^	1		1	
BMI > 24 kg/m^2^	0.97(0.74–1.26)	0.810	0.97(0.74–1.27)	0.830
Nonsmoking	1		1	
Smoking	1.11(0.85–1.44)	0.454	1.04(0.79–1.38)	0.777
Nondrinking	1		1	
Drinking	0.96(0.74–1.24)	0.742	0.76(0.58–0.99)	0.044
Normotensive	1		1	
Hypertension	1.97(1.51–2.56)	<0.001	2.61(1.98–3.43)	<0.001
Normal blood lipids	1		1	
Hyperlipidemia	1.56(0.89–1.50)	0.264	1.36(1.04–1.78)	0.024
Non-diabetes	1		1	
Diabetes	1.95(1.39–2.72)	<0.001	1.39(0.98–1.98)	0.064

*CAD* coronary artery disease, *IS* ischemic stroke

### *MAFB* SNPs and serum lipid levels

The significant association was found between the genotypes of the rs2902940 SNP and serum ApoAI levels in the controls (*P* = 0.018), but not in the CAD and IS patients (Table 6). The subjects with the GA/AA genotype in controls had a decreased serum ApoAI level as compared with the GG genotype (*P* = 0.024). No significant association was found between the genotypes of the rs6102059 SNP and serum lipid levels (*P* > 0.05).

**Table 6:** Effect of the genotypes on serum lipid levels

Group/Genotype	TC (mmol/L)	TG (mmol/L)	HDL-C (mmol/L)	LDL-C (mmol/L)	ApoAI (g/L)	ApoB (g/L)	ApoAI/ApoB
Control							
rs2902940							
GG	4.64 ± 0.14	1.10(0.93)	1.56 ± 0.06	2.74 ± 0.11	1.24 ± 0.03	0.88 ± 0.03	1.45 ± 0.21
GA	4.78 ± 0.08	1.16(0.96)	1.47 ± 0.03	2.82 ± 0.06	1.19 ± 0.02	0.89 ± 0.02	1.56 ± 0.12
AA	4.63 ± 0.08	1.01(0.67)	1.46 ± 0.03	2.77 ± 0.06	1.14 ± 0.02	0.90 ± 0.02	1.35 ± 0.11
*P*	0.346	0.222	0.312	0.765	0.018	0.838	0.440
GG	4.64 ± 0.14	1.10(0.93)	1.56 ± 0.06	2.74 ± 0.11	1.24 ± 0.03	0.88 ± 0.03	1.45 ± 0.21
GA + AA	4.71 ± 0.05	1.08(0.79)	1.45 ± 0.02	2.79 ± 0.04	1.16 ± 0.01	0.89 ± 0.01	1.45 ± 0.08
*P*	0.673	0.680	0.136	0.653	0.024	0.615	0.986
rs6102059							
CC	4.70 ± 0.11	1.13(0.85)	1.45 ± 0.04	2.78 ± 0.08	1.14 ± 0.03	0.89 ± 0.02	1.29 ± 0.16
CT	4.69 ± 0.08	1.02(0.78)	1.48 ± 0.03	2.77 ± 0.06	1.17 ± 0.02	0.89 ± 0.02	1.55 ± 0.11
TT	4.71 ± 0.09	1.13(0.85)	1.51 ± 0.04	2.81 ± 0.07	1.20 ± 0.02	0.90 ± 0.02	1.41 ± 0.14
*P*	0.976	0.919	0.593	0.928	0.235	0.857	0.396
CC	4.70 ± 0.11	1.13(0.85)	1.45 ± 0.04	2.78 ± 0.08	1.14 ± 0.03	0.89 ± 0.02	1.29 ± 0.16
CT + TT	4.70 ± 0.06	1.05(0.81)	1.49 ± 0.02	2.78 ± 0.05	1.18 ± 0.01	0.89 ± 0.01	1.50 ± 0.09
*P*	0.943	0.733	0.427	0.971	0.146	0.972	0.268
CAD							
rs2902940							
GG	4.60 ± 0.17	1.12(0.59)	1.39 ± 0.07	2.59 ± 0.13	1.16 ± 0.04	0.87 ± 0.04	1.39 ± 0.10
GA	4.67 ± 0.08	1.18(0.80)	1.49 ± 0.03	2.75 ± 0.06	1.17 ± 0.02	0.91 ± 0.02	1.41 ± 0.04
AA	4.56 ± 0.07	1.23(0.83)	1.41 ± 0.03	2.70 ± 0.06	1.14 ± 0.02	0.90 ± 0.02	1.34 ± 0.04
*P*	0.608	0.440	0.153	0.486	0.511	0.509	0.440
GG	4.60 ± 0.17	1.12(0.59)	1.39 ± 0.07	2.59 ± 0.13	1.16 ± 0.04	0.87 ± 0.04	1.39 ± 0.10
GA + AA	4.61 ± 0.05	1.21(0.80)	1.45 ± 0.02	2.73 ± 0.04	1.15 ± 0.01	0.90 ± 0.01	1.37 ± 0.03
*P*	0.954	0.317	0.427	0.307	0.919	0.312	0.877
rs6102059							
CC	4.64 ± 0.11	1.18(0.81)	1.46 ± 0.04	2.76 ± 0.08	1.16 ± 0.03	0.90 ± 0.02	1.37 ± 0.06
CT	4.56 ± 0.07	1.17(0.66)	1.45 ± 0.03	2.65 ± 0.06	1.15 ± 0.02	0.89 ± 0.02	1.40 ± 0.04
TT	4.66 ± 0.10	1.33(1.00)	1.42 ± 0.04	2.80 ± 0.08	1.15 ± 0.02	0.92 ± 0.02	1.32 ± 0.06
*P*	0.687	0.066	0.763	0.248	0.923	0.317	0.510
CC	4.64 ± 0.11	1.18(0.81)	1.46 ± 0.04	2.76 ± 0.08	1.16 ± 0.03	0.90 ± 0.02	1.37 ± 0.06
CT + TT	4.60 ± 0.06	1.21(0.79)	1.44 ± 0.02	2.70 ± 0.05	1.15 ± 0.01	0.90 ± 0.01	1.37 ± 0.03
*P*	0.718	0.359	0.690	0.536	0.738	0.852	0.969
IS							
rs2902940							
GG	4.63 ± 0.17	1.32(0.99)	1.19 ± 0.06	2.73 ± 0.13	1.02 ± 0.03	0.94 ± 0.04	1.11 ± 0.09
GA	4.51 ± 0.07	1.41(0.95)	1.22 ± 0.03	2.68 ± 0.06	1.03 ± 0.02	0.90 ± 0.02	1.18 ± 0.04
AA	4.51 ± 0.07	1.27(0.91)	1.24 ± 0.03	2.67 ± 0.06	1.02 ± 0.02	0.88 ± 0.02	1.17 ± 0.04
*P*	0.799	0.480	0.581	0.928	0.884	0.235	0.751
GG	4.63 ± 0.17	1.32(0.99)	1.19 ± 0.06	2.73 ± 0.13	1.02 ± 0.03	0.94 ± 0.04	1.11 ± 0.09
GA + AA	4.51 ± 0.05	1.36(0.93)	1.23 ± 0.02	2.68 ± 0.04	1.02 ± 0.01	0.89 ± 0.01	1.18 ± 0.03
*P*	0.503	0.765	0.496	0.703	0.967	0.147	0.501
rs6102059							
CC	4.53 ± 0.11	1.27(1.06)	1.22 ± 0.04	2.68 ± 0.09	1.01 ± 0.02	0.90 ± 0.02	1.20 ± 0.06
CT	4.52 ± 0.07	1.39(0.93)	1.21 ± 0.02	2.68 ± 0.05	1.02 ± 0.01	0.89 ± 0.02	1.16 ± 0.04
TT	4.52 ± 0.09	1.34(0.88)	1.26 ± 0.03	2.69 ± 0.07	1.03 ± 0.02	0.89 ± 0.02	1.16 ± 0.05
*P*	0.997	0.324	0.372	0.992	0.844	0.906	0.823
CC	4.53 ± 0.11	1.27(1.06)	1.22 ± 0.04	2.68 ± 0.09	1.01 ± 0.02	0.90 ± 0.02	1.20 ± 0.06
CT + TT	4.52 ± 0.06	1.37(0.92)	1.23 ± 0.02	2.68 ± 0.04	1.03 ± 0.01	0.89 ± 0.01	1.16 ± 0.03
*P*	0.943	0.210	0.913	0.941	0.632	0.664	0.533

The value of triglyceride was presented as median (interquartile range) and used nonparametric Test

*TC* total cholesterol, *TG* triglyceride, *HDL*-*C* high-density lipoprotein cholesterol, *LDL*-*C* low-density lipoprotein cholesterol, *ApoAI* apolipoprotein *AI*, *ApoB* apolipoprotein B *CAD* coronary artery disease, *IS* ischemic stroke

## Discussion

In the present study, we selected two loci in the *MAFB* to perform genetic association analysis in the Chinese Han population. To the best of our knowledge, it is the first report to evaluate the association between the *MAFB* SNPs and serum lipid levels and the risk of CAD and IS in the Chinese population.

Genetic association studies in humans have been informative for investigating the role of *MAFB* SNPs in serum lipid levels [13, 14]. In a recent meta-analysis, Teslovich et al. [13] reported that the minor G allele of rs2902940 conferred a higher level of serum LDL-C and a lower level of serum TC in European descent. In another study, Kathiresan et al. [14] have shown an association between the *MAFB* rs6102059 SNP and serum LDL-C levels in European descent. The minor T allele of rs6102059 was negatively related to the level of serum LDL-C. *MAFB* was showed to interact with LDL-related protein [23]. Petersen et al. [23] reported that the transcription factor *MAFB*, as novel the LDL receptor-related protein interacting protein, interacted with the intracellular domain of LDL receptor-related protein through a leucine zipper domain and negatively regulated LDL receptor transcriptional activity, suggesting that *MAFB* was likely to influence lipid metabolism, and the variants in *MAFB* destroyed this interaction and changed serum lipid levels. However, inconsistent with previous studies, our research did not discover significant correlations between the *MAFB* SNPs and serum LDL-C and TC levels. Nevertheless, after correction for multiple testing, our findings showed that there was significant association between the genotypes of the rs2902940 SNP and serum ApoAI levels in the controls, but not in the CAD and IS patients. The subjects with rs2902940 GA/AA genotype had lower levels of serum ApoAI than those with GG genotype. However, the rs6102059 SNP showed no apparent effect in serum lipid levels. The reason for these diverse findings is not clear and may be due to different genetic background. In our study, the frequency of rs2902940 G and A alleles was 34.6 and 65.4 %, and rs6102059 C and T alleles was 44.5 and 54.5 % in health controls, which somewhat differed with the data from the International HapMap project: the G and A allele frequencies of rs2902940 were 27.4 and 72.6 %, and the C and T allele frequencies of rs6102059 were 71.7 and 28.3 % in European descent. On the other hand, relatively little is known about the function of *MAFB* on lipid, thereby, further investigated is needed to underatand the function of the gene.

In the present study, we showed that the AA genotype of rs2902940 SNP was associated with an increased risk of CAD and IS. Multivariate analysis showed that known risk factors, such as hypertension, diabetes and hyperlipidemia, were independently associated with CAD and IS. Additionally, the rs2902940 GA/AA genotypes were also associated with an increased risk of CAD and IS after adjusting for potential confounding factors. In the stratified analysis, an increased risk of CAD and IS in subjects with the GA/AA genotype was mainly observed in males, aged ≤ 60, drinkers, BMI > 24 kg/m^2^ and those with hypertension and diabetes. In addition, an increased risk of IS in subjects with the GA/AA genotype was also noted in smokers, and those with normal blood lipids. Significant interactions were observed only in those with BMI > 24 kg/m^2^, hypertension and diabetes, suggesting gene-environment interactions contributed to the risk of CAD and IS. Recent studies showed that *MAFB* was involved in macrophage apoptosis, which was important in the development of atherosclerosis [19], suggesting a role of *MAFB* in the disease. Macrophage foam cells play a critical role in the occurrence and development of atherosclerosis. Formation of macrophage foam cells in the intima is a major hallmark of early stage atherosclerotic lesions [5]. In the early stages of atherosclerosis, uncontrolled uptake of oxidized low-density lipoprotein (ox-LDL) in the intima induces dysfunction of endothelial cells and smooth muscle cells, leading to the production of proinflammatory cytokines including chemokines that recruit monocytes. These recruited monocytes become macrophages that take up ox-LDL via scavenger receptors. Then, the pathogenic macrophages transform into foam cells and form the fatty streak, which constitutes the early atherogenic lesion and ultimately triggers the more advanced atherosclerotic plaques. The accelerated apoptosis of foam cells could lead to reduced lesion size and a subsequent attenuation of plaque progression [24, 25]. *MAFB* was reported to predominantly express in foam cells and enhanced their survival in aortic atherosclerotic lesions, where *MAFB* regulated the heterodimerized nuclear receptor liver X receptor (LXR)/retinoid X receptor (RXR)-induced expression of apoptosis inhibitor of macrophages (AIM) [19]. In the absence of *MAFB*, activated LXR/RXR failed to induce the expression of AIM, a member of the scavenger receptor cysteine-rich superfamily that was normally responsible for protecting macrophages from apoptosis [26, 27], thus, *MAFB*-deficient macrophages were prone to apoptosis [19]. Haematopoietic reconstitution with *MAFB*-deficient fetal liver cells in recipient LDL receptor-deficient (LDLRˉ^/^ˉ) hyperlipidemic mice led to accelerating foam-cell apoptosis, which subsequently caused the attenuation of the early atherogenic lesion [19]. The macrophage-affiliated *MAFB* transcription factor participated in the acceleration of atherogenesis by exerting an anti-apoptotic function in macrophages. Therefore, we could infer that the *MAFB* variants might play a deleterious role in human vascular biology and favor the incidence of CAD and IS. In the present study, we showed that the A allele carriers of SNP rs2902940 in the *MAFB* had a decreased serum ApoAI level and were also associated with an increased risk of CAD and IS. Thus, it was possible that the rs2902940 SNP directly altered the function of *MAFB* or, alternatively, was in LD with the other causative genetic variants which could be related to CAD and IS. Although the rs2902940 SNP linked to an increased risk of CAD, we could not find the association between the rs2902940 SNP and angiographic severity of CAD, suggesting that the pathogenic effects of this gene were unlikely to be a major pathway for higher CAD and IS risk, but subtle effects cannot be excluded.

In addition, it is well known that *MAFB* plays a critical role in regulating the essential functions of developing, differentiating and establishing the function of cells, tissues and organs [28–30], including pancreatic islets [28]. Genome scans in families with type 2 diabetes identified a putative locus on chromosome 20q, *MAFB* was found with islet expressed sequence tags, and showed relatively high expression [31]. *MAFB* played critical roles in α and β cell differentiation [28, 32]. In the pancreas, *MAFB* binded directly to regulate sequences containing the G1 element in the endogenous glucagon gene and activated glucagon gene transcription [33]. *MAFB* interacted with other pancreatic transcription factors to induce the expression of insulin and other key β cell markers [28]. It is well known that diabetes was independently risk factor for incidence of CAD and IS, and it may interact with the SNP of rs2902940 in the *MAFB* to favor the incidence of CAD and IS. Thus, *MAFB* was considered to be involved in the regulation of glucose metabolism, which consequently contributes to development of CAD and IS. Together, the effects of *MAFB* gene on lipid metabolism and atherosclerosis are complex and multiple channels needed further research.

The present study has two potential limitations. First, the impact of drug could not be fully evaluated or excluded. The IS patient groups had lower levels of serum TC and the ratio of ApoAI to ApoB, and the genotype of the rs2902940 SNP was associated with serum ApoAI levels in the controls, but not in the CAD and IS patients, which might be related to using lipid-lowing drugs in the patient groups. Second, the two SNPs did not cover the whole gene cluster and could not overall explain the associations of *MAFB* polymorphisms with the diseases. Therefore, the observed associations need further replications to avoid spurious associations.

## Conclusions

The results of the present study showed that there was significant association between the *MAFB* rs2902940 SNP and the risk of CAD and IS. The *MAFB* rs2902940A allele carriers conferred a decreased serum ApoAI level and an increased risk of CAD and IS. The GA/AA genotypes interacted with higher BMI, hypertension and diabetes to contribute the risk of CAD and IS. Our findings should be important to help clarifying the role of *MAFB* in atherosclerosis.

## Methods

### Study population

A total of 1,065 unrelated patients with CAD (*n* = 525) and IS (*n* = 540) were recruited from hospitalized patients in the First Affiliated Hospital, Guangxi Medical University. The diagnosis of CAD was based on typical clinical symptoms and electrocardiographic changes, as well as increases in the serum markers including creatinine kinase-MB and troponin T. Coronary angiography was performed in patients with CAD. The selected CAD patients were subject to significant coronary stenosis (≥50 %) in at least either one of the three main coronary arteries or their major branches (branch diameter ≥ 2 mm). Additionally, angiographic severity of disease was defined as single or multi-vessel disease based on the number of involved artery (luminal narrowing ≥ 50 %) in the three major coronary arteries [34, 35]. The classification of IS was made according to the TOAST (Trial of Org 10172 in Acute Stroke Treatment) criteria [36]. The selected IS patients in the study included individuals who were eligible for one of the two subtypes of TOAST criteria: Large-artery atherosclerosis and small-vessel occlusion. Subjects with a history of hematologic, neoplastic, renal, liver, thyroid, autoimmune diseases and type I diabetes mellitus were excluded. The selected IS patients who had a past history of CAD were excluded, while the selected CAD patients who had a past history of IS were excluded from the study.

A total of 539 control subjects matched by age, gender, and ethnic group (Han Chinese) were consecutively recruited from Physical Examination Center of the First Affiliated Hospital, Guangxi Medical University during the same period when IS and CAD patients were recruited. The controls were free of IS and CAD by questionnaires, history taking and clinical examination. All enrolled individuals were Han Chinese from Guangxi, the People’s Republic of China. A standard questionnaire was used to ascertain the general information and medical history for all participants. The study protocol was approved by the Ethics Committee of the First Affiliated Hospital, Guangxi Medical University. Informed consent was obtained from all subjects after they received a full explanation of the study.

### Biochemical measurements

Venous blood sample was obtained from all subjects after at least 12 h of fasting. The levels of serum TC, TG, HDL-C, and LDL-C in samples were determined by enzymatic methods with commercially available kits. Serum ApoAI and ApoB levels were detected by the immunoturbidimetric immunoassay. The normal values of serum TC, TG, HDL-C, LDL-C, ApoAI, ApoB levels, and the ratio of ApoAI to ApoB in our Clinical Science Experiment Center were 3.10–5.17, 0.56–1.70, 0.91–1.81, 2.70–3.20 mmol/L, 1.00–1.78, 0.63–1.14 g/L, and 1.00–2.50; respectively [37–39]. The individuals with TC > 5.17 mmol/L, and/or TG > 1.70 mmol/L were defined as hyperlipidemic [40]. Hypertension was diagnosed according to the criteria of the JNC 7 hypertension guidelines [41]. Uncontrolled hypertension was defined as a systolic blood pressure of 140 mmHg or higher and a diastolic blood pressure of 90 mmHg or higher. The subjects with systolic blood pressure of only 140 mmHg or higher but a diastolic blood pressure of < 90 mmHg were diagnosed as isolated systolic hypertension. Normal weight, overweight and obesity were defined as a BMI < 24, 24–28, and > 28 kg/m^2^; respectively [42].

### SNP selection and genotyping

The SNPs of rs2902940 and rs6102059 were selected as genetic markers. The two SNPs were selected on the basis of the following assumptions: (1) Selected SNPs were established by Haploview (Broad Institute of MIT and Harvard, USA, version 4.2); (2) SNPs information was obtained from NCBI dbSNP Build 132 (http://www.Ncbi.nlm.nih.gov/SNP/); (3) SNPs were restricted to minor allele frequency (MAF) > 1 %. (4) SNPs might be associated with the plasma lipid levels in a recent GWAS [13, 14].

Genomic DNA was extracted from leucocytes of venous blood using the phenol-chloroform method, and then sent to the Center for Human Genetics Research, Shanghai Genesky Bio-Tech Co. Ltd. Genotyping of the two SNPs (rs2902940, rs6102059) were performed by the Snapshot technology platform. The restriction enzymes for loci rs2902940, rs6102059 were SAP and EXO I (Promega, Epicentre), respectively. The sense and antisense primers were: rs2902940F: 5′-CCGCAGGCTGTCTCAGGTTTTA-3′, rs2902940R: 5′-AAGGCAAATAGGGAGTAGAAAAACACTTG-3′; rs6102059F: 5′-TCTGCTAAGCCSTTTATGTGTACCATCT-3′,rs6102059R: 5′-TGACATTCCCAGGGGTGGACT-3′.

### Statistical analyses

The statistical analyses were carried out using the statistical software package SPSS 21.0 (SPSS Inc., Chicago, Illinois). Quantitative variables were expressed as mean ± standard deviation (serum TG levels were presented as medians and interquartile ranges). Qualitative variables were expressed as percentages. Allele frequency was determined via direct counting, and the standard goodness-of-fit test was used to test the Handy-Weinberg equilibrium. A chi-square analysis was used to evaluate the difference in genotype distribution and sex ratio between the groups. The general characteristics between patients and controls were tested by the Student’s unpaired *t*-test. The association of genotypes and serum lipid parameters was tested by analysis of covariance (ANCOVA). Any variants associated with the serum lipid parameter at a value of *P* < 0.025 (corresponding to *P* < 0.05 after adjusting for two independent tests by the Bonferroni correction) were considered statistically significant. Unconditional logistic regression was used to assess the correlation between the risk of CAD and IS and genotypes. Age, gender, BMI, smoking and alcohol consumption were adjusted for the statistical analysis. OR and 95 % CI were calculated using unconditional logistic regression. Results were considered to be statistically significant if bilateral *P*-values were less than 0.05. The pattern of pair-wise LD between the selected SNPs was measured by D’ and *r*^2^ using the SHEsis software [43].

## Abbreviations

AIM: Apoptosis inhibitor of macrophages
ANCOVA: Analysis of covariance
Apo: Apolipoprotein
BMI: Body mass index
bZIP: Basic leucine-zipper
CAD: Coronary artery disease
CI: Confidence interval
DALYs: Disability-adjusted life years
DNA: Deoxyribonucleic acid
GWAS: Genome-wide association studies
HDL-C: High-density lipoprotein cholesterol
IS: Ischemic stroke
LD: Linkage disequilibrium
LDL-C: Low-density lipoprotein cholesterol
LDLR: Low-density lipoprotein receptor
LXR: Liver X receptor
MAF: Minor allele frequency
OR: Odds ratio
ox-LDL: Oxidized low-density lipoprotein
RXR: Retinoid X receptor
SNP: Single nucleotide polymorphism
T2DM: Type 2 diabetes mellitus
TC: Total cholesterol
TG: Triglyceride
TOAST: Trial of Org 10172 in Acute Stroke Treatment
